# Diagnosis of hereditary hemorrhagic telangiectasia based on endoscopic detection of oral telangiectasias

**DOI:** 10.1055/a-2641-1743

**Published:** 2025-07-25

**Authors:** Masaya Iwamuro, Kenta Hamada, Motoyuki Otsuka

**Affiliations:** 1 12997 Department of Gastroenterology and Hepatology, Okayama University Graduate School of Medicine, Dentistry, and Pharmaceutical Sciences, Okayama, Japan

A 58-year-old man presented with dizziness and a three-month history of recurrent epistaxis. Laboratory tests revealed severe microcytic anemia (hemoglobin: 5.3 g/dL), prompting hospitalization and blood transfusion. His medical history included hypertension and hyperlipidemia. Notably, his mother also had a history of frequent nosebleeds. Upper gastrointestinal endoscopy was performed to investigate potential gastrointestinal bleeding. Esophagogastroduodenoscopy revealed multiple reddish areas on the tongue ( Video 1 , Fig. 1 , arrows). Small red dots were observed on the palate, consistent with telangiectasia ( Fig. 2 , arrow). Additionally, reddish lesions were observed at the base of the tongue ( Fig. 3 , arrow). These findings suggested hereditary hemorrhagic telangiectasia (HHT). Scattered telangiectasias were identified in the stomach, further supporting the diagnosis ( Fig. 4 , arrows). Contrast-enhanced computed tomography revealed a contrast-enhancing hepatic lesion, likely a hemangioma ( Fig. 5 , arrow). However, no arteriovenous malformations were observed in the lungs or brain.

**Fig. 1:**
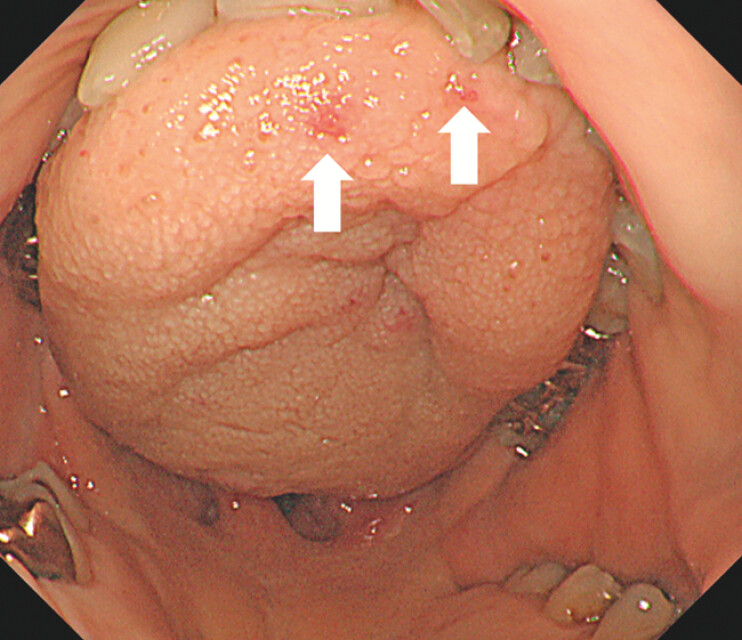
Esophagogastroduodenoscopy showing multiple reddish areas on the tongue (arrows).

**Fig. 2:**
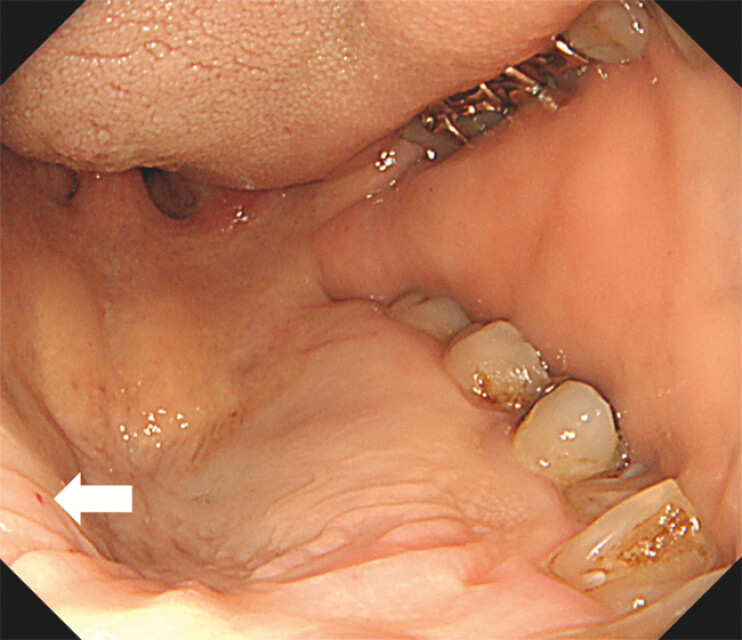
Esophagogastroduodenoscopy showing small red dots on the palate (arrow).

**Fig. 3:**
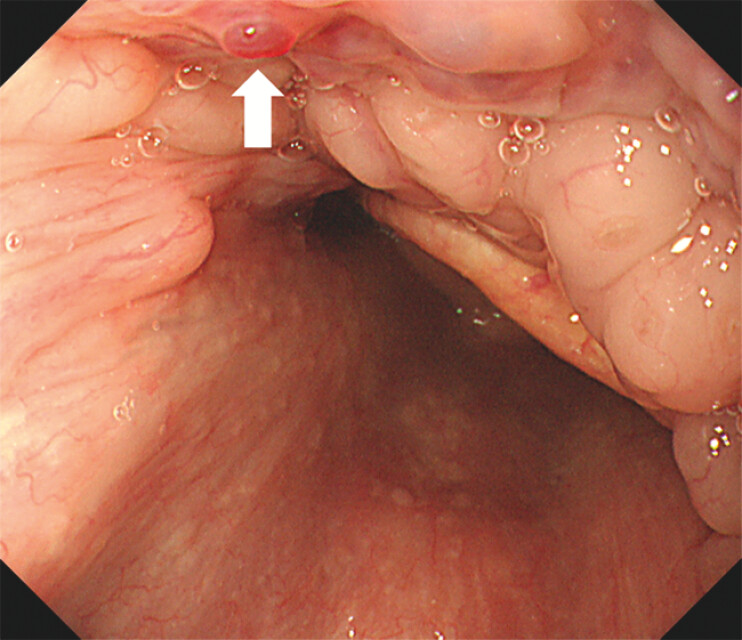
Esophagogastroduodenoscopy showing blood blister-like protruded lesions at the base of the tongue (arrow).

**Fig. 4:**
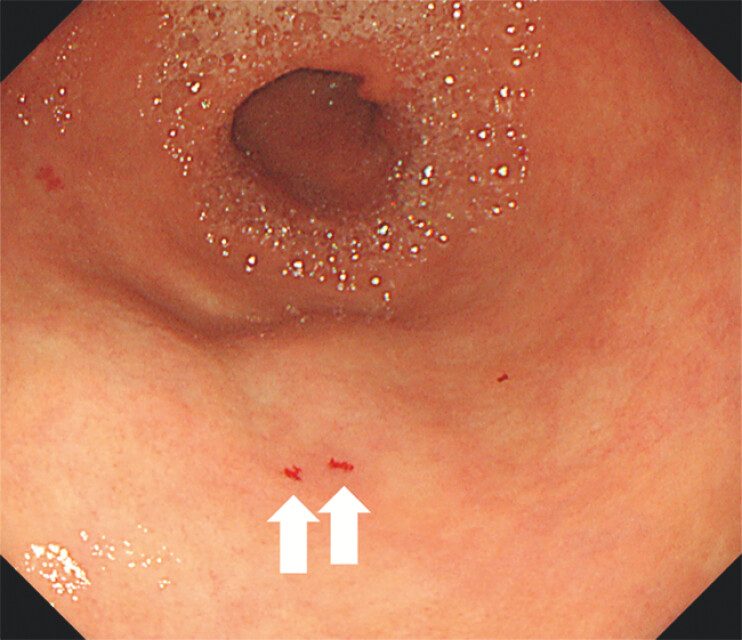
Esophagogastroduodenoscopy showing telangiectasia in the stomach (arrows).

**Fig. 5:**
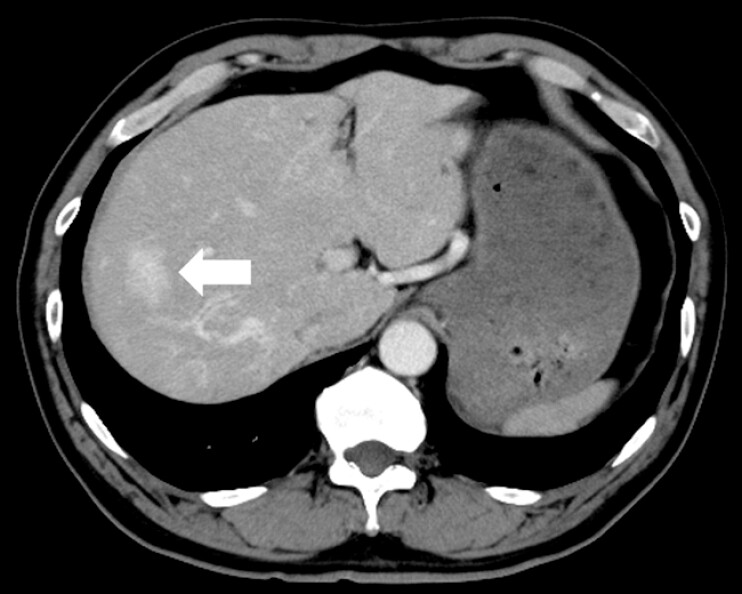
Contrast-enhanced computed tomography showing a hepatic mass with peripheral contrast enhancement, suspected to be a hemangioma.

Video of oral observation via esophagogastroduodenoscopy, showing reddish lesions on the tongue and palate.Video 1

The patient fulfilled the three Curaçao diagnostic criteria for HHT 1 : recurrent epistaxis, mucocutaneous telangiectasia, and visceral involvement (gastric telangiectasia and hepatic lesions). Although the patient’s family history could not be definitively confirmed, his mother’s history of frequent epistaxis was suggestive of a hereditary component.

HHT is an autosomal-dominant vascular disorder characterized by mucocutaneous telangiectasias and arteriovenous malformations in various organs, including the lungs, brain, liver, and gastrointestinal tract. Clinical manifestations vary widely and may lead to life-threatening complications, such as hemorrhage, stroke, or high-output cardiac failure, if left undiagnosed 2 3 4 . Therefore, early recognition is essential to prevent serious outcomes through appropriate surveillance and management of arteriovenous malformations. This case highlights the diagnostic value of oral endoscopic findings in patients with HHT, leading to further systemic evaluation. Careful inspection of the oral cavity, including the tongue and palate, in patients with recurrent epistaxis can facilitate early diagnosis and timely management of this multisystem vascular disorder.

Endoscopy_UCTN_Code_CCL_1AB_2AB

## Notes

Endoscopy E-Videos https://eref.thieme.de/e-videos
*E-Videos* is an open access online section of the journal *Endoscopy* , reporting on interesting cases and new techniques in gastroenterological endoscopy. All papers include a high-quality video and are published with a Creative Commons CC-BY license. Endoscopy E-Videos qualify for HINARI discounts and waivers and eligibility is automatically checked during the submission process. We grant 100% waivers to articles whose corresponding authors are based in Group A countries and 50% waivers to those who are based in Group B countries as classified by Research4Life (see: https://www.research4life.org/access/eligibility/ ). This section has its own submission website at https://mc.manuscriptcentral.com/e-videos .
